# The Relationship between Fatigue and Cytokine Levels in Patients with Acute Myeloid Leukemia

**Published:** 2018-10-01

**Authors:** Malihe Khosravi, Hamidreza Taghvaye Masoumi, Kheirollah Gholami, Mohammad Vaezi, Molouk Hadjibabaei, Ardeshir Ghavamzadeh

**Affiliations:** 1 Hematology-Oncology and Stem Cell Transplantation Research Center, Tehran University of Medical Sciences, Tehran, Iran; 2 Department of Clinical Pharmacy, School of Pharmacy, Tehran University of Medical Sciences, Tehran, Iran

**Keywords:** Cytokines, Cancer-related fatigue, Acute myeloid leukemia

## Abstract

**Background:** Cancer-related fatigue (CRF) is a very prominent complaint and disabling symptom in cancer patients probably influenced by endogenous cytokines. But, the published data on this subject are limited. We explored the relationship of cytokines such as tumor necrosis factor (TNF-α) and interleukin-6 (IL-6) with fatigue in patients with AML.

**Materials and Methods:** This study was performed on 45 patients (25 men, 20 women) with newly diagnosed AML. We examined fatigue in these patients with validated questionnaire. Simultaneously, blood samples were obtained for quantitative measurement of IL-6 and TNF-α.

**Results:** Our results showed a positive correlation between fatigue and circulating levels of IL-6 (P=0.004, R=0.416).

**Conclusion: **Many patients with AML experienced severe fatigue before the onset of treatment, which is not related to their hemoglobin (Hb) levels. Cytokine levels may be beneficial markers in resistance to fatigue, but further studies are needed before considering targeted therapies as a treatment for CRF.

## Introduction

 Acute myeloid leukemia (AML) is a group of hematopoietic malignancies which associated with the involvement of the myeloid precursor cells. Consequently, an accumulation of non-functional myeloid cells (the so-called leukemic blasts) occurs in the bone marrow which interferes with the production of normal blood cells^1^. AML is the most common acute leukemia in adults which occurs more frequently in elderly patients. 

Cytokines are a broad class of small proteins secreted by specific cells that are important in cell communications. Interleukins (ILs) are a group of cytokines with different functions such as the effects on proliferation, differentiation and activation of immune cells^2^.

Cancer-related fatigue (CRF) is a more severe form of fatigue that can be experienced at any stage of the disease even before cancer diagnosis and usually persists long after treatment and is not attenuated by adequate sleep or rest. It can affect the quality of life of patients ^3^^-^^7^. 

In addition to the complications of chemotherapy drugs, CRF is a disabling symptom in AML patients. There is no definite definition for CRF, but it is multifactorial and the underlying etiological factors, including weight loss, anemia, fever, pain, mood and sleep disturbance, infection and thyroid disorder^6^^,^^8^^,^^9^. Surprisingly, CRF associated with AML, unlike solid cancer-related fatigue, is not related to anemia^7^^,^^10^. Moreover, there are significant relationships between psychological factors such as anxiety and depression with fatigue^11^.

The main aim of the present study was to evaluate the relationship between pro-inflammatory cytokines (IL-6 and TNF-α) and fatigue in patients with AML.

## MATERIALS AND METHODS

 This prospective observational study was carried out between September 2016 and June 2017 at the Hematology–Oncology and Stem Cell Transplantation Research Center (HOSCRC), Shariati Hospital, Tehran University of Medical Sciences, Tehran, Iran. The study was approved by the local Ethics Committee of the institution. Written informed consent was obtained from all patients at the beginning of the study (Ethics code: IR.TUMS.PSRC.REC.1395.1603).

Adult patients (≥18 years) with newly diagnosed AML undergoing 7+3 induction chemotherapy (IC) regimen were enrolled in the study. Subjects were excluded from the study if they had AML M3, myelodysplastic syndrome (MDS), immune system disorders, severe infection during the previous week, secondary AML or relapsed AML. None of the patients had previously been treated by chemotherapy agents. AML diagnosis based on bone marrow aspiration/ biopsy and pathological results were confirmed by a hematologist at Shariati Hospital. The present study assessed the relationship between pro-inflammatory cytokine levels (IL-6 and TNF-α) and fatigue.

All patients were evaluated for fatigue on day before receiving 7 + 3 chemotherapy regimen according to the Functional Assessment of Chronic Illness Therapy-Fatigue (FACIT-Fatigue) Scale (Version 4) ^9^. This scale is a 13-item questionnaire that has a maximum of 52 points. The higher score represents the better quality of life. A score of less than 30 represents severe fatigue.

The 7+3 chemotherapy regimen was used as IC in all patients and consisted of 7 days of continuous intravenous (IV) infusion of standard-dose cytarabine (100 mg/m^2^/day) and 3 days of short infusion (30 min) of daunorubicin (60 mg/m^2^/day).

Five milliliters (ml) venous blood samples were collected on the day before starting IC. The samples were collected in ethylenediaminetetraacetic acid (EDTA) tubes and centrifuged (3000 rpm, 4°C, 10 min) immediately after collection. Subsequently, the separated plasma was moved to a polypropylene micro tube and stored at −80°C until the time of assay. The plasma levels of TNF-α and IL-6 were measured by high sensitivity enzyme-linked immunosorbent assay (ELISA) kits (IBL INTERNATIONAL GMBH, Hamburg, Germany) according to the manufacturer's instructions.

Statistical analyses were performed using SPSS V.14 statistical software. Descriptive statistics were expressed as mean ± standard deviation (SD) or median for quantitative variables and frequency (percentage) for qualitative variables. The relationship between cytokine concentration and fatigue was evaluated by the Spearman’s rank test. In our study, P ≤ 0.05 was considered statistically significant.

## Results

 A total of 45 eligible patients with a mean age of 42.62 ±12.21 years (range: 18-65) were enrolled in the study. The patient's demographic data, disease information and laboratory parameters are presented in Table 1.

**Figure 1:**
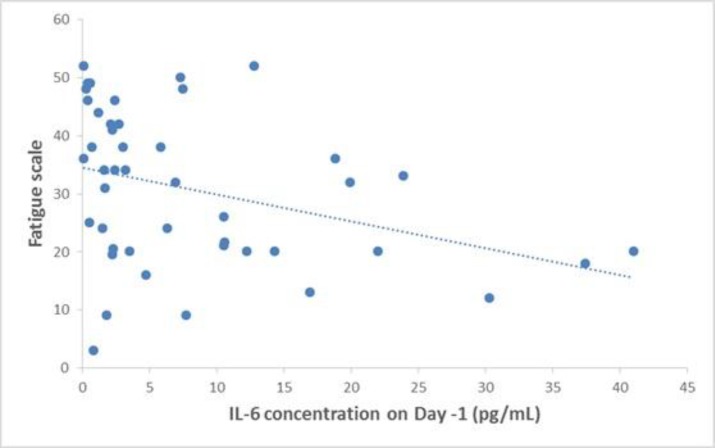
Correlation between IL-6 and fatigue scale in patients with AML non M3. The higher serum concentrations of IL-6 correlated with more severe fatigue (P=0.004, R=0.416).

Twenty (44.4%) patients had severe fatigue (score <30). The mean fatigue scale was 30.81 ± 13.22 (range: 3-52). Our results showed that only IL-6 demonstrated a significant correlation with FACT-F, so that patients with higher serum concentrations of IL-6 had more severe fatigue (P=0.004, R=0.416). This relationship is shown in Figure 1.

Of 45 patients, 22 (48.8%) achieved CR at the end of first cycle of chemotherapy. There was no significant relationship between fatigue scale and CR rate.

**Table 1:** Demographic, Clinical and Laboratory Data of Patients

**Variable **	** Patients**
Age (years)	42.62 ±12.21 (range:18-65)
Gender (women/men)	20/25
Weight (kg)	69.69 ±12.97 (range: 39-104)
AML type M_0_ M_1_ M_2_ M_3_ M_4_ M_5_ M_6_ M_7_	3 10 14 0 13 4 1 0
Response at 1 month CR No CR	22 (48.89%) 23(51.11%)
IL-6 concentration (pg/mL)	3.2 (range: 0.1-41)
TNF-α concentration (pg/mL)	0.9 (range: 0.1-16.3)

## Discussion

 The relationship between pro-inflammatory cytokines and fatigue was investigated in the current study. In our study, the only cytokine that was correlated with fatigue was IL-6. The higher plasma levels of IL-6 were associated with more severe fatigue (or a lower fatigue scale).

Our findings are consistent with Schubert et al. and Meyers et al. who reported a correlation between IL-6 and fatigue ^6^^,^^12^. According to Meyers et al., IL-6 levels were significantly associated with fatigue and overall quality of life ratings. Also, higher IL-6 levels were associated with poorer performance for executive function^12^. Some studies show that fatigue in breast cancer was associated with higher levels of IL-6 ^13^^,^^14^. Liu et al. found that both fatigue and IL-6 levels increased concomitantly with chemotherapy in breast cancer patients^13^.

There are other studies that support the relationship between fatigue and inflammation, for example, Mills et al. revealed the relationship of fatigue with soluble intercellular adhesion molecule-1 (sICAM-1) and vascular endothelial growth factor (VEGF) in breast cancer patients^15^. Based on the results mentioned above, it is possible that cytokines may play an important role in pathophysiology of cancer-related fatigue as a common biological basis.

To improve the quality of life among patients with cancer, it is necessary to investigate CRF and its correlation with inflammatory cytokines to clarify the biological mechanisms of fatigue and to promote the development of targeted interventions.

However, the results should be interpreted with caution due to the relatively small sample size.

This study had certain limitations. In addition to the relatively small sample size, fatigue was evaluated only before the onset of IC and the study was performed using only a few cytokines.

In conclusion, data from the present study showed which cytokines were only one of the several contributing factors in CRF. Further studies are needed to confirm this subject.
